# Edaravone Exerts Protective Effects on Mice Intestinal Injury without Interfering with the Anti-Tumor Effects of Radiation

**DOI:** 10.3390/cimb45070340

**Published:** 2023-06-28

**Authors:** Terufumi Kawamoto, Keisuke Sasai

**Affiliations:** Department of Radiation Oncology, Graduate School of Medicine, Juntendo University, Tokyo 113-8421, Japan

**Keywords:** enteritis, radiation, radioprotector, radiotherapy

## Abstract

The appropriate dosage of edaravone—a radioprotective agent—and its effect on tumors are unknown. This study evaluated the effects of edaravone on intestinal injuries and tumors in mice induced by whole body X-ray irradiation. Small intestinal mucositis was induced in C3H/HeNSlc mice using a single X-ray dose (15 Gy). Edaravone (15, 30, and 100 mg/kg) was administered 30 min before irradiation to evaluate its protective effect. After 3.5 days, the jejunum was removed and the histological changes were evaluated. Next, C3H/HeNSlc mice with squamous cell carcinoma VII tumors were provided the same single X-ray dose and 100 mg/kg edaravone; further, the tumors were immediately induced after irradiation. The tumor cell viability was detected using an in vivo–in vitro colony formation assay. We found that the intestinal colony-forming ability after irradiation was significantly higher in the 100 mg/kg edaravone group than that in the control group. Moreover, the apoptotic cells in the villi immunohistochemically stained with cleaved caspase-3 were significantly lower in the 100 mg/kg edaravone group than in the control group. We found no radioprotective effects of intraperitoneally inoculated edaravone in both hind legs on squamous cell carcinoma VII tumors. These findings suggest that 100 mg/kg edaravone exerts protective effects on small intestinal injuries without interfering with the antitumor effects of radiation.

## 1. Introduction

Cancer is one of the leading causes of death worldwide, accounting for an estimated 10.0 million deaths in 2020 [1]. Owing to recent advances in diagnostic and treatment techniques, approximately 50% of patients with cancer receive radiotherapy (RT) during the disease course [2]. Over the past century, progress in RT has prolonged survival and improved the control of disease and treatment-related toxicities [3].

Remarkably, ionizing RT causes the radiolysis of water molecules in cells and produces reactive oxygen species (ROS). Approximately 70% of the biological damage due to RT is caused by ROS [4]. Stereotactic body radiotherapy (SBRT) is a modern irradiation technique that delivers high doses of radiation to a tumor via highly conformal techniques [5]. Moreover, it is a noninvasive technique performed on an outpatient basis and used to treat lesions in the lungs, liver, kidneys, bones, oligometastatic lesions, etc. However, severe intestinal toxicity has been reported during the irradiation of organs with large internal motion, such as in cases of pancreatic cancer [6]. The major clinical manifestations of intestinal toxicity are frequent stools and diarrhea in addition to severe manifestations, such as tenesmus and intestinal perforation [7]. In some cases, these toxicities require hospitalization and can impact the patient’s quality of life. Current treatment modalities for radiation enteritis are symptomatic and focus on oral therapy to control bowel movements, and there is inadequate evidence to strongly support the utility of prophylactic medication before RT [8]. Bagheri et al. showed that metformin could be used as a radioprotective for the small intestine, which can be due to the suppression of mitochondria production by reactive oxygen species or its action on inflammatory pathways [9]. However, the metformin presented in this paper have established preclinical data as radioprotective agents for the small intestine but lack human testing. Implants or expanders have been used to reduce the small intestinal toxicity; Sugarbaker et al. described their use to exclude the small intestine from the pelvic cavity [10]. However, these devices have to be adapted to the pelvic space because vein compression, giving rise to deep venous thrombosis and pulmonary embolism has been reported [11].

Notably, antioxidants have been widely used to protect cells from the damage caused by ROS. In particular, edaravone—which is chemically represented as 3-methyl-1-phenyl-2-pyrazolin-5-one—is widely used to treat acute cerebral infarction and amyotrophic lateral sclerosis (ALS). In addition, it is a scavenger of several ROS and may exert radioprotective effects [12]. Anzai et al. reported that edaravone has a radioprotective effect against lethal whole body X-ray irradiation in mice [13]. Furthermore, in previous basic studies, edaravone reduced radiation-induced hippocampal apoptosis, oral mucositis, and ovarian damage [14,15,16]. However, the efficacy and appropriate doses of edaravone to reduce radiation-induced small intestinal injuries are unclear.

Antioxidant drugs have produced inconsistent results for tumors, and some clinical and basic studies indicated that antioxidants may increase the tumor risk [17,18,19]. However, to the best of our knowledge, no studies have examined its effects on edaravone’s interference with the antitumor effects of RT. Therefore, this study aimed to evaluate the effect of edaravone on mice with small intestine injuries and tumors induced by high-dose X-ray irradiation.

## 2. Materials and Methods

### 2.1. Animals

Overall, 33 8–10-week-old C3H/HeNSlc female mice (20 g; Sankyo Labo Service Corporation Inc., Tokyo, Japan) were used for all experiments. These mice were housed in a room with a temperature of 22 ± 2 °C and humidity of approximately 55%, maintaining a 12-h light–dark cycle. They were fed a standard rodent diet and had free access to water. Furthermore, the general health parameters of the mice were assessed in terms of body weight. Those exhibiting >20% body weight loss after 1 week of rearing were excluded from the experiment.

The animal experimental protocol was followed in accordance with the guidelines by the Ethics Review Committee for Animal Experimentation of Juntendo University (Registration number: 1245).

### 2.2. Drugs

In humans, 30 mg of edaravone (3-methyl-1-phenyl-2-pyrazolin-5-one; Radicut^®^) was intravenously infused. Notably, edaravone was provided by Mitsubishi Tanabe Pharma Corporation (Osaka, Japan). It was dissolved in a small volume of 1 M NaOH solution. The pH of the solution was adjusted to 7 using 1 M HCL in accordance with the relevant previous studies [16,20]. The prepared edaravone was used as a solution. Further, the concentration was adjusted to 1.5, 3, or 10 mg/mL in 0.9% (physiologic) saline solution in accordance with the relevant previous studies [15,21,22]. Previous studies reported that 15, 30, and 100 mg/kg edaravone can improve ALS, radiation-induced oral mucositis, and cisplatin-induced renal injury, respectively [15,21,22]. Subsequently, 15, 30, or 100 mg/kg edaravone was injected intraperitoneally 30 min (min) before irradiation. In contrast, saline was injected in the control group.

### 2.3. Radiation

**Figure 1:**
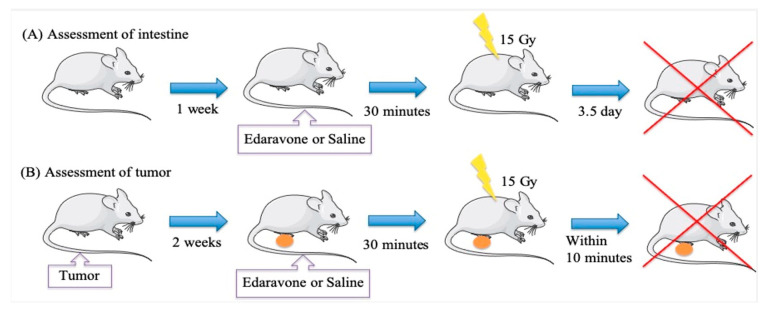
Schematic representation of the experiment. (**A**) Mice were fed for 1 week as an acclimation period (days 1–7). Next, edaravone or saline was intraperitoneally administered 30 min before administering irradiation. The mice were irradiated on day 8, fed until day 10, and euthanized on day 11. (**B**) Mice were subcutaneously inoculated with SCC VII cells on day 1. Mice were fed for 2 weeks until the growth of tumor was observed (days 1–14). In addition, edaravone or saline was intraperitoneally administered 30 min before administering irradiation. Mice were irradiated and euthanized on day 15.

Figure 1 presents an experimental overview.

Irradiation was performed using a device (MBR-1520R2; Hitachi, Tokyo, Japan) with a voltage of 150 kV and a current of 5 mA at a dose rate of 1.5 Gy/min using an Al 0–5 mm filter. Mice were placed in a special gauge (150 mm × 150 mm × 50 mm) and irradiated with a single whole body dose of 15 Gy without anesthesia. Remarkably, the single whole body dose of 15 Gy was determined based on a relevant previous study [23]. The conditions of the irradiation room where the experiment was conducted were such that they could prevent the radiation from contaminating other areas of the laboratory.

### 2.4. Experimental Design

This study comprised two parts: assessment of the intestine and tumor.

For the intestinal assessment (in vivo), 33 mice were classified into 6 groups in the laboratory. In group 1 (n = 5; intact), saline was administered, and no irradiation was provided throughout the study period. In group 2 (n = 5; control), saline was administered 30 min before irradiation, and 15 Gy whole body irradiation was provided. In group 3 (n = 6), 15 mg/kg edaravone was intraperitoneally administered 30 min before irradiation, and 15 Gy whole body irradiation was provided. In group 4 (n = 6), 30 mg/kg edaravone was intraperitoneally administered 30 min before irradiation, and 15 Gy whole body radiation was provided. In group 5 (n = 6), 100 mg/kg edaravone was intraperitoneally administered 30 min before irradiation, and 15 Gy whole body radiation was provided. In group 6 (n = 5), 100 mg/kg edaravone was administered intraperitoneally, and no irradiation was applied throughout the study. Notably, all mice were euthanized via cervical dislocation after 3.5 days of irradiation in accordance with Withers’s method (Figure 1) [24]. No anesthesia or analgesics were used for the mice. Nevertheless, to alleviate suffering, they were euthanized via cervical dislocation with a humane endpoint of ≥20% weight loss after irradiation. Notably, the changes in weight were evaluated before and 3.5 days after irradiation. The jejunum was removed, and it was fixed in formalin and evaluated. Further, histopathological changes and immunoreactivity for cleaved caspase 3 were noted and compared between the groups under light microscopy.

To assess the tumor, we used SCC VII tumor cells (squamous cell-like cells kindly provided by Professor Miura, Tokyo Medical and Dental University, Tokyo, Japan) of C3H/He mice. The SCC VII cells were then cultured in minimal essential medium (Thermo Fisher Scientific, Waltham, MA, USA) containing 10% fetal bovine serum (Sigma-Aldrich, St. Louis, MO, USA) and 1% penicillin–streptomycin solution (Sigma-Aldrich). These cells were collected from monolayer cultures, and approximately 1 × 10^5^ cells were subcutaneously inoculated in both hind legs of the 12 syngeneic female mice. After 14 days, the volume of tumors reached 200 mm^3^ (experimental size). Subsequently, saline or 100 mg/kg edaravone was intraperitoneally administered 30 min before irradiation, and 15 Gy whole body radiation or no irradiation was applied. Twelve mice were classified into four groups in the laboratory. In group A (n = 3; intact), saline was administered, and no Irradiation was provided throughout the study period. In group B (n = 3; control), saline was administered 30 min before irradiation, and 15 Gy whole body irradiation was provided. In group C (n = 3), 100 mg/kg edaravone was intraperitoneally administered 30 min before irradiation, and 15 Gy whole body radiation was provided. In group D (n = 3), 100 mg/kg edaravone was administered intraperitoneally, and no irradiation was applied throughout the study. The mice were then euthanized via cervical dislocation within 10 min of irradiation (Figure 1). The surviving fractions were determined using an in vivo–in vitro colony assay, as described previously [25]. In summary, the mice were euthanized within 10 min of irradiation, and the tumors were excised, minced, and magnetically stirred with 0.05% trypsin and 0.02% EDTA in Hanks’ balanced salt solution (Thermo Fisher Scientific) for 15 min at 37 °C to obtain single-cell suspensions. Further, the known appropriate numbers of cells were plated in tissue culture dishes with a diameter of 6 cm. These colonies were fixed in 70% ethanol after 10–11 days and stained with crystal violet (Fujifilm Wako Pure Chemical Corporation, Osaka, Japan) dissolved in 70% ethanol. Notably, colonies containing >50 cells were scored as survivors.

### 2.5. Histopathological Examination

The specimens of the intestinal tissue were fixed in 10% neutral-buffered formalin, dehydrated, and embedded in paraffin. Paraffin sections were stained with hematoxylin–eosin (HE) and observed under light microscopy. The renewal process of the intestine involves the rapid and continuous proliferation of epithelial cells in the crypt base with subsequent migration of these cells along the crypt–villus axis [26]. Thus, the number of surviving intestinal crypts per circumference in the transverse section of the intestine was counted. Further, ten transverse sections of the intestine were randomly selected. The intestinal colony-forming ability was determined by the formula S = −ln((A − x)/A) [24], where S represented the survival of crypts per circumference, A represented the number of crypts per circumference in the transverse section of the intestine of the non-irradiated group (group 1), and x represented the number of crypts per circumference in the transverse section of the intestine in the irradiated group.

The degree of intestinal tissue injury was evaluated on a scale of 0–8 using the Park’s injury score for the following 10 transverse sections: 0, normal mucosa; 1, subepithelial space at the villus tip; 2, more extended subepithelial space; 3, epithelial lifting along villus sides; 4, denuded villi; 5, loss of villus tissue; 6, crypt layer infarction; 7, transmucosal infarction; and 8, transmural infarction [27].

### 2.6. Immunohistochemistry

Immunohistochemistry was performed in accordance with Shibata’s method [28,29]. The anticleaved caspase-3 rabbit antibody (No. 9664, 1:50; Cell Signaling Technology Japan, K.K., Tokyo, Japan) was used for immunohistochemistry. Notably, ROS released from irradiation directly damaged cells to induce apoptosis and crypt hypoplasia [30]. Notably, Caspase-3—the key enzyme involved in apoptosis in the small intestine—is cleaved and activated by other caspases to yield cleaved caspase 3 [31]. The positive cell count was calculated as follows. First, fields were randomly selected, and pictures were taken under 400× magnification of a microscope. Second, 10 villi were randomly selected for the analysis. Finally, the percentage of positive cells to the total cells composing each villus was manually calculated.

### 2.7. Statistical Analyses

To compare the study groups, we used the nonparametric one-way Kruskal–Wallis ANOVA followed by post hoc analysis for multiple comparisons [32]. All statistical analyses were performed using EZR version 1.54 [33], and *p*-values of <0.05 (two-sided) were considered statistically significant.

### 2.8. All Materials Details

- C3H/HeNSlc mice (Sankyo Labo Service Corporation Inc., Tokyo, Japan)
- Edaravone (3-methyl-1-phenyl-2-pyrazolin-5-one; Radicut^®^; Mitsubishi Tanabe Pharma Corporation, Osaka, Japan)
- Radiation device (MBR-1520R2; Hitachi, Tokyo, Japan)
- SCC VII tumor cells (squamous cell-like cells provided by Professor Miura, Tokyo Medical and Dental University, Tokyo, Japan)
- Medium (Thermo Fisher Scientific, Waltham, MA, USA)
- 10% fetal bovine serum (Sigma-Aldrich, St. Louis, MO, USA)
- 1% penicillin–streptomycin solution (Sigma-Aldrich, St. Louis, MO, USA)
- Hanks’ balanced salt solution (Thermo Fisher Scientific, Waltham, MA, USA)
- Crystal violet (Fujifilm Wako Pure Chemical Corporation, Osaka, Japan)
- Anticleaved caspase-3 rabbit antibody (No. 9664, 1:50; Cell Signaling Technology Japan, K.K., Tokyo, Japan)

## 3. Results

### 3.1. Body Weight

We found that the changes in body weight (standard error) before and 3.5 days after irradiation were −0.075 (0.29), −3.6 (0.2), −3.5 (0.2), −3.48 (0.18), −3.2 (0.11), and 0.1 (0.27) g for groups 1, 2, 3, 4, 5, and 6, respectively.

### 3.2. Histopathological Results

**Figure 2:**
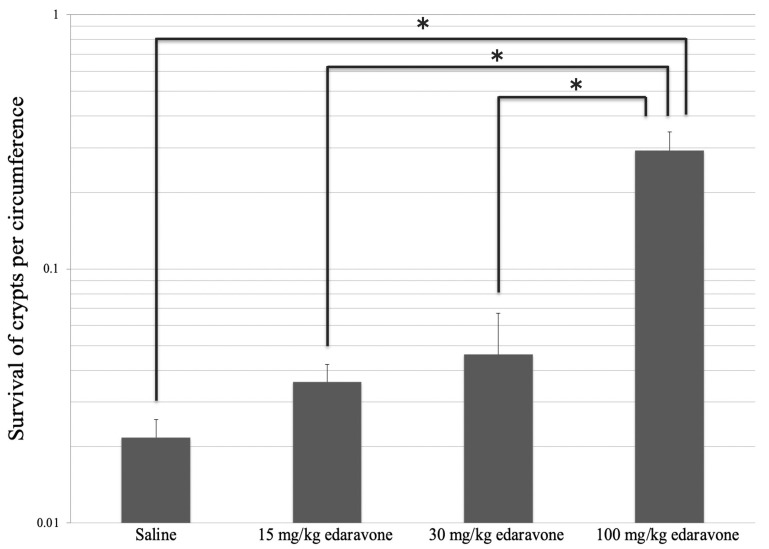
Effect of administration of edaravone before irradiation. The intestinal colony-forming ability after irradiation was significantly higher in the 100 mg/kg edaravone group (group 5) (* *p* < 0.05).

Notably, the values for the intestinal colony-forming ability (standard error) after irradiation were 0.022 (0.0039), 0.036 (0.006), 0.046 (0.021), and 0.29 (0.054) for groups 2, 3, 4, and 5, respectively (Figure 2). The Kruskal-Wallis test confirmed the high intestinal colony-forming ability of the mice in group 5 (*p* = 0.0013). Post hoc analyses revealed that a significant difference was found between groups 2 and 5, 3 and 5, and 4 and 5 (*p* = 0.031, 0.021, and 0.021, respectively).

The Park’s injury mean scores (standard error) were 0.12 (0.048), 5.74 (0.085), 5.43 (0.14), 5.35 (0.51), 3.62 (0.39), and 0.15 (0.029) for groups 1, 2, 3, 4, 5, and 6, respectively. A significant difference was found between groups 2 and 5 (*p* = 0.008).

**Figure 3:**
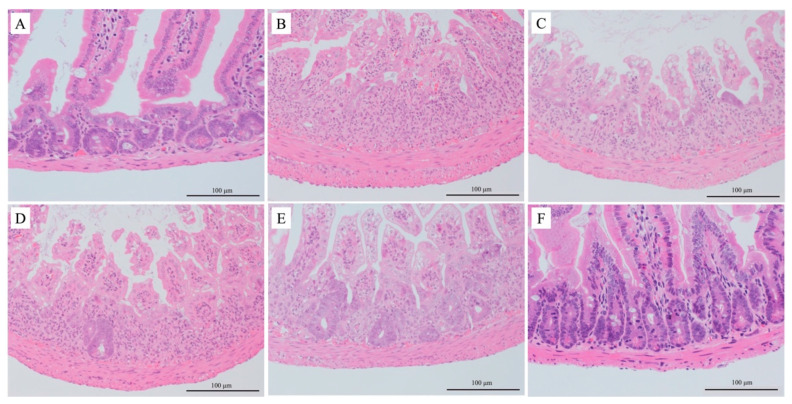
Histopathological evaluation via hematoxylin and eosin staining. (**A**) Saline and non-irradiated group (group 1); (**B**) saline and irradiated group (group 2); (**C**) 15 mg/kg edaravone and irradiated group (group 3); (**D**) 30 mg/kg edaravone and irradiated group (group 4); (**E**) 100 mg/kg edaravone and irradiated group (group 5); and (**F**) 100 mg/kg edaravone and non-irradiated group (group 6).

In the no irradiation group (groups 1 and 6), the number of viable crypts was similar between the groups receiving saline and edaravone administration. In the irradiation group (groups 2–5), the number of viable crypts was preserved in the 100 mg/kg edaravone group (Figure 3).

### 3.3. Immunohistochemical Results

**Figure 4:**
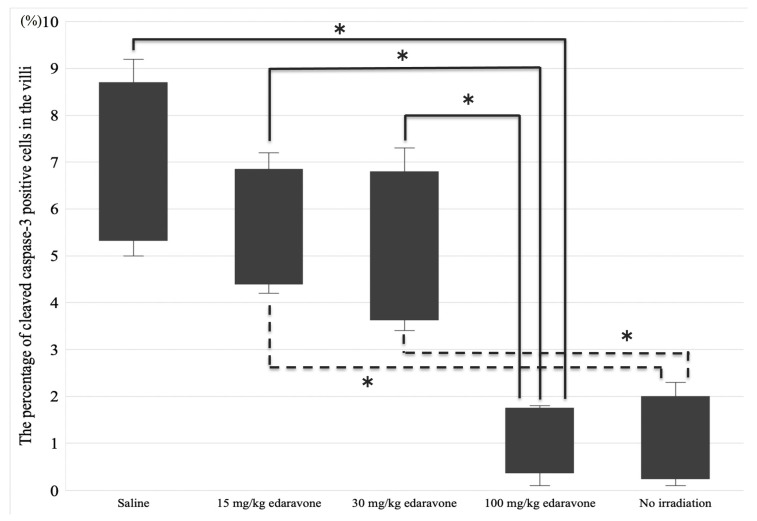
Percentage of cleaved caspase-3-positive cells. The apoptotic cells in the villi were significantly lower in the 100 mg/kg edaravone group (group 5) (* *p* < 0.05).

The percentages of cleaved caspase-3-positive cells (standard error) in the villi were 1.1% (0.46), 6.9% (0.88), 5.6% (0.63), 5.1% (0.84), and 1.2% (0.38) for groups 1, 2, 3, 4, and 5, respectively (Figure 4). The Kruskal-Wallis test confirmed the low percentages of cleaved caspase-3-positive cells in group 5 (*p* < 0.001). Post hoc analysis revealed that a significant difference was found between groups 2 and 5, 3 and 5, 4 and 5, 1 and 3, and 1 and 4 (*p* = 0.049, 0.032, 0.032, 0.049, and 0.049, respectively).

**Figure 5:**
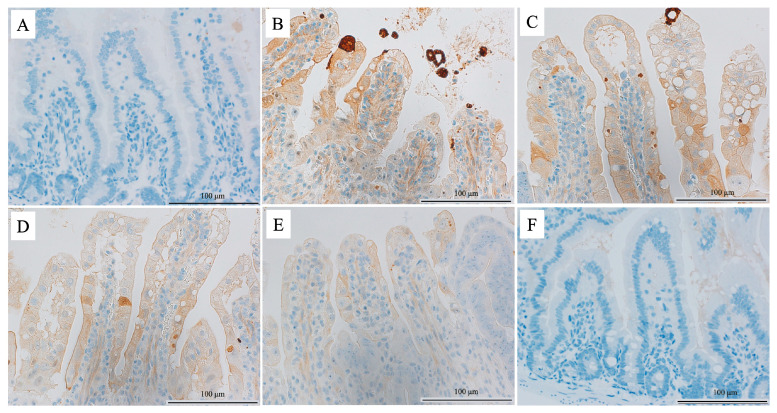
Immunohistochemical evaluation of apoptosis-related cleaved caspase-3. (**A**) Saline and non-irradiated group (group 1); (**B**) saline and irradiated group (group 2); (**C**) 15 mg/kg edaravone and irradiated group (group 3); (**D**) 30 mg/kg edaravone and irradiated group (group 4); (**E**) 100 mg/kg edaravone and irradiated group (group 5); and (**F**) 100 mg/kg edaravone and non-irradiated group (group 6).

In the no-irradiation group (groups 1 and 6), the cleaved caspase-3-positive cells in the villi were similar between the saline and 100 mg/kg edaravone groups. In contrast, in the irradiation group (groups 2–5), the cleaved caspase-3-positive cells in the villi were higher in the saline group than in the 100 mg/kg edaravone group (Figure 5).

### 3.4. Effects of Edaravone on Mice Tumor

**Figure 6:**
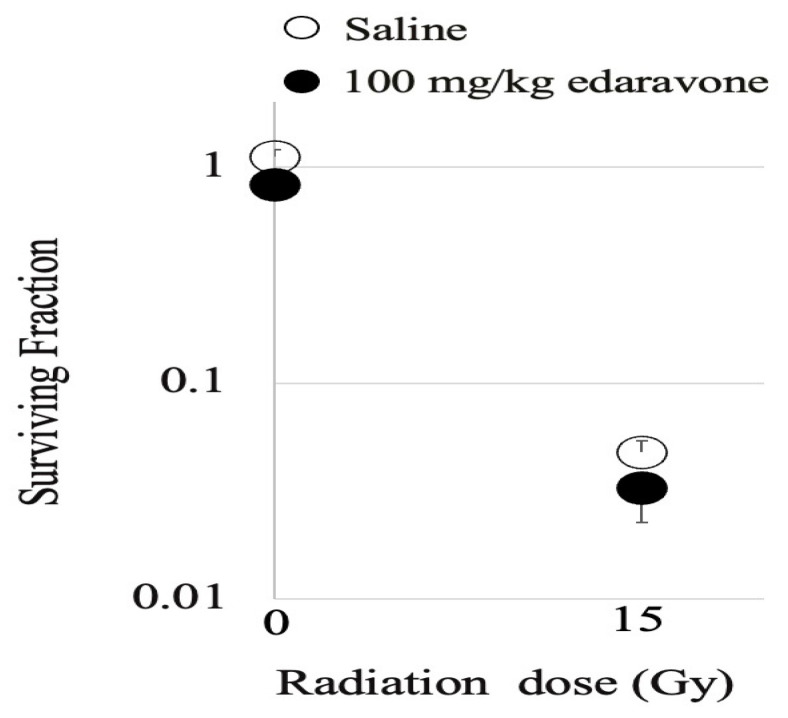
Surviving fraction of SCC VII tumor cells after a single dose of 15 Gy to mice treated with normal saline (open circle) or 100 mg/kg edaravone (closed circle). Values represent the means of ≥3 samples in three independent experiments; error bars indicate standard deviations.

An intraperitoneal injection of 100 mg/kg edaravone, which represents the same treatment as that of the intestinal assay, did not demonstrate any radioprotective effects on SCC VII tumors (Figure 6).

## 4. Discussion

This study confirmed that 100 mg/kg edaravone significantly reduces small intestinal injuries observed after 3.5 days without interfering with the antitumor effects of radiation.

Notably, edaravone is a low-molecular-weight agent that readily crosses the blood–brain barrier; therefore, its activity is not limited to the vascular compartment [34] In addition to its direct antioxidant activity, edaravone exerts other effects that might make it useful for treating several non-neurological diseases and clinical conditions. Regarding radiation injury, some basic studies have reported that edaravone reduces radiation-induced hippocampal apoptosis, oral mucositis, and ovarian injury [14,15,16]. Moreover, it reduced ischemia/reperfusion-induced small intestinal injuries in mice [35]. Thus, it can be potentially used in various organs and associated conditions. However, these doses of edaravone are much higher than those that have been historically used in humans.

Ito et al. reported the effective plasma concentrations of edaravone for brain ischemia in experiments involving animals and older adults. Notably, a daily dose of 15 mg/kg intraperitoneally administered edaravone was found to be suitable for the ALS model mice [21]. Nakajima et al. reported that 30 and 300 mg/kg edaravone reduced the levels of radiation-induced oral mucositis, myeloperoxidase activity, and apoptosis rate after comparing the results of terminal deoxynucleotidyl transferase-mediated deoxyuridine triphosphate-biotin nick-end label staining results with the control group [15]. Koike et al. reported that 100 mg/kg of edaravone led to a better improvement of cisplatin-induced renal injury in the experimental group than in the control group [22]. Based on these reports, we set the intraperitoneal doses of edaravone for radiation-induced small intestinal injury model mice at 15, 30, and 100 mg/kg. In our study, the intestinal colony-forming ability after irradiation was significantly higher in the 100 mg/kg edaravone group than that in the control group, whereas no significant difference was found in the 15 and 30 mg/kg edaravone groups. These findings suggest that the administration of edaravone above a certain dose is necessary to prevent radiation injuries.

In clinical practice, edaravone is indicated for cerebral infarction and ALS. Edaravone was administered at a dose of 30 mg twice per day for 14 days based on the dosage used in patients with cerebral infarction [36]. Only one prospective randomized clinical trial has evaluated the protective effect of edaravone on radiation-induced brain necrosis in patients with nasopharyngeal carcinoma [37]. The use of the same dose of edaravone in patients with cerebral infarction led to a significant improvement in MRI-detected edema as well as neurologic symptoms and signs. This result suggested that the same dose of edaravone used in daily clinical practice may prevent radiation-induced enteritis and oral mucositis in addition to brain necrosis.

During the planning of the present experiment, we thought that edaravone could interfere with the antitumor effects of radiation. In contrast to this expectation, interference with the antitumor effects was not observed. Furthermore, the activation of the nuclear factor kappa B (NF-kB) in tumor cells was found to attenuate chemotherapy- or RT-induced apoptosis [38]. A previous study reported that edaravone did not affect the antitumor effects of chemotherapy by inhibiting the activation of NF-kB [39]. Although no study has reported RT’s antitumor effects on edaravone to the best of our knowledge, our study findings suggest that edaravone does not interfere with the antitumor effects of RT by inhibiting the activation of NF-kB.

This study has some limitations. First, the sample size of the animals was small, which might have influenced the statistical analysis. However, a previous study observed the effect of radiation enteritis in similar sample sizes [23], and the enteritis radioprotective effect was considered sufficient for this study. Second, the antitumor and enteritis protective effects were not confirmed in the same participants at the same time points. Furthermore, the process of previous experiments was considered difficult because of the different timings of euthanizing mice and evaluating samples.

## 5. Conclusions

We found that edaravone significantly reduces radiation-induced small intestine injuries in mouse models. In addition, it does not interfere with the antitumor effects of radiation. These results were based on therapeutic strategies using edaravone that could support the RT administration in clinical practice.

Additional prospective studies are warranted to evaluate the effects of edaravone in clinical settings and determine whether edaravone is beneficial for radiation-induced toxicities.

## Data Availability

The data presented in this study are available upon request from the corresponding author.
